# Inadequate Protein Intake at Specific Meals Is Associated with Higher Risk of Impaired Functionality in Middle to Older Aged Mexican Adults

**DOI:** 10.1155/2019/6597617

**Published:** 2019-04-04

**Authors:** Alejandro Gaytán-González, María de Jesús Ocampo-Alfaro, Maritza Arroniz-Rivera, Francisco Torres-Naranjo, Roberto Gabriel González-Mendoza, Martha Gil-Barreiro, Juan Ricardo López-Taylor

**Affiliations:** ^1^Institute of Applied Sciences for Physical Activity and Sport, Department of Human Movement Sciences, Education, Sport, Recreation and Dance, University Health Sciences Center, University of Guadalajara, Guadalajara, Jalisco, Mexico; ^2^Department of Human Reproduction, Infantile Growth and Development, University Health Sciences Center, University of Guadalajara, Guadalajara, Jalisco, Mexico; ^3^Geriatrics Department, Western General Hospital, Zapopan, Jalisco, Mexico; ^4^Center of Body Composition and Bone Research, Guadalajara, Jalisco, Mexico

## Abstract

**Purpose:**

To describe the proportions of inadequate protein intake (IPI) per day and per meal and their association with functionality in middle to older aged Mexican adults.

**Materials and Methods:**

In a cross-sectional design, we evaluated the protein intake and functionality of instrumental activities of daily living (IADL) and activities of daily living (ADL) of 190 middle to older aged Mexican adults. IPI was considered as any protein intake: <1.2 g/kg/day, <30 g/meal, or <0.4 g/kg/meal. Functionality was organized into three groups: high, middle, and low scores. The first was set as the reference, and the other was considered as impaired functionality. With a multinomial logistic regression, we analyzed the association between IPI per day and per meal with impaired functionality.

**Results:**

A high proportion of participants showed IPI per day. The meal with the highest proportion of IPI was dinner, followed by breakfast and lunch for both criteria. IPI at lunch was a significant risk factor for impaired functionality in ADL when assessed with the 30 g/meal criterion (low scores, OR 3.82 (95% CI, 1.15–12.65); middle scores, OR 2.40 [1.03–5.62]). For the 0.4 g/kg/meal criterion, IPI at dinner was a significant risk factor for IADL middle scores only (OR 7.64, [1.27–45.85]).

**Conclusion:**

IPI per meal is high in middle to older aged Mexican adults, and at specific meals, it is a significant risk factor for impaired functionality in activities of daily living.

## 1. Introduction

Sarcopenia was initially deemed as the age-related decrease in muscle mass [1–3]; nowadays, the term also encompasses loss of muscle strength [2–5] and impaired functionality [6–8]. However, sarcopenia has been redefined recently, where muscle strength is the primary criterion to consider probable sarcopenia, which is confirmed by low muscle mass and functionality used to classify its severity [9]. These conditions are of interest because any one of them is related to a lower quality of life [10–12] and increased mortality [7, 13, 14].

While resistance exercise has been shown to have a robust effect on age-related declines in muscle mass and strength [15], adequate daily and per meal protein intake is associated with higher lean muscle mass and strength in older adults [16–18]. Current evidence [19, 20] suggests that older adults should consume protein at a dosage of at least 1.0–1.2 g/kg/day to preserve or even increase muscle mass [21–23]. It is also suggested that older adults should consume protein at a dosage ≥30 g per meal [20, 24] or ≥0.4 g/kg per meal [25] to adequately stimulate muscle protein synthesis.

The interest in how much protein older adults consume and whether this amount is adequate or not has increased recently. Several studies have reported a high proportion of older adults consuming protein below the RDA [26, 27], the recommended dosage of 1.0 g/kg/day [28–30], 30 g of protein per meal [24, 27], and 0.4 g/kg per meal [29, 30] in different countries. These reports suggest that a high percentage of the elderly population may be at higher risk for developing sarcopenia and its related complications [31]. Currently, however, data regarding habitual protein intake in middle to older aged Mexican adults and its association with functionality in activities of daily living is scarce [27]. Therefore, the purpose of this study was to describe the proportions of inadequate protein intake (IPI) per day and per meal and their association with functionality in middle to older aged Mexican adults.

## 2. Materials and Methods

### 2.1. Study Design and Participants

This was a cross-sectional study where we evaluated the protein intake and functionality of middle to older aged Mexican adults attending the Department of Geriatrics at the Western General Hospital (Hospital General de Occidente. Zapopan, Jalisco, Mexico) from January to July 2017 for their usual medical screening or their initial assessment. All assessments were evaluated once (the first visit within the recruitment period).

Subjects were included if they fulfilled the following criteria: (1) they were aged 50 years or older; (2) they were able to answer the questionnaires independently (minimal assistance of their caregivers was permitted if necessary), and (3) they were able to stand up and walk unassisted (only canes were allowed). Subjects were not eligible if they reported any kind of hospitalization within the last year. We excluded cases for the analysis if participants were unable to provide detailed dietary information or if data were incomplete. This convenience nonprobabilistic sample was initially composed of 659 possible participants, but 191 did not meet the last two inclusion criteria and 278 were excluded (258 did not provide detailed nutritional data, and 20 were discarded for missing data), leading to 190 participants (141 women, 49 men) aged 53 to 97 years. We evaluated all participants for all assessments after they were informed about the study objectives, procedures, and possible risks, and we obtained a signed written statement of consent before any procedure was performed. The Institutional Review Board of the University Health Sciences Center from the University of Guadalajara approved this protocol.

### 2.2. General Data Assessment

We obtained clinical data employing the standard case file required by law [32]. This documentation includes data about sex, age, diagnosed diseases, body weight (to nearest 0.5 kg), height (to nearest 1 cm), BMI, and BMI categories for the elderly. For anthropometric measurements, subjects wore light clothing and no shoes. BMI was calculated as body mass (kg) divided by height squared (m^2^). BMI was categorized as recommended (23–30.9 kg/m^2^), below (<23) the recommended, and above (≥31) the recommended for older adults [33].

### 2.3. Functionality

Participants answered two validated questionnaires to assess their functionality. The first was the Lawton questionnaire to evaluate the instrumental activities of daily living (IADL) [34]. The second was the Barthel questionnaire to evaluate the activities of daily living (ADL) [35]. One of the investigators, who directly interviewed the participant, administered both questionnaires, and the caregiver's help was allowed. These tools consisted of several items (IADL, eight for women and five for men; ADL, ten for all) that evaluated the participants' ability to successfully perform some daily activities that involve employing tools for self-maintenance and living in the community (e.g., handling finances, using the toilet, and independent transportation). The items were coded as “functional” if participants answered the item according to the authors' scale for success [34, 35] and as “impaired” if they did not. Afterward, we categorized the final score as low, middle, and high if men reported 0-1, 2-3, or 4-5 scores and women 0–2, 3–5, or 6–8 for the IADL, respectively. For the ADL, the ranges were 0–60, 61–90, or 91–100 for low, middle, and high scores, respectively [36]. The higher the score, the more functional the subject. Previous studies reported good internal consistency for both questionnaires (Cronbach's alpha: Lawton 0.94; Barthel 0.82) and a significant association with health-related quality of life [37, 38].

### 2.4. Dietary Intake and Inadequate Protein Intake Assessment

We evaluated participants' dietary intake with a 24 h dietary recall performed by trained nutritionists following standardized procedures [39]. Briefly, nutritionists followed the US Department of Agriculture's multistep methodology to collect detailed information about the protein-rich foods that participants consumed in the recorded day. This method consists of five steps: (1) record the foods as the participant reminds them; (2) complete the previous foods with commonly forgotten food (e.g., salads, sauces, and sweeteners); (3) categorize the foods into specific meals; (4) describe in detail the ingested foods (preparation, brands, and amount); and (5) review the recorded information to confirm it was correctly reported and there is no something else to report [39]. Food replicas were used for enhancing the estimation of food servings.

Then, one external researcher analyzed all dietary recalls to estimate the amount of ingested protein at every main meal (breakfast, lunch, and dinner) and per day, employing a specialized software (Nutrickal® VO v1.1, Ogali-COSINFO SC, Mexico). This external researcher test-retest error was 2.2% for breakfast, 3.3% for lunch, 2.4% for dinner, and 1.9% for total protein intake. Protein intake per meal and per day was expressed as absolute (g/day, g/meal) and relative to body mass (g/kg/day, g/kg/meal).

Inadequate protein intake (IPI) was considered as any protein intake placed below the recommended amount of 1.2 g/kg/day [20] and in a per meal basis as doses beneath 30 g/meal [24] or 0.4 g/kg/meal [25]. For informative purposes, we calculated the IPI for the protein RDA (0.8 g/kg/day) and for the lower bound of the protein recommendation (1.0 g/kg/day) [20]. These two statistics were not employed for further statistical analyses.

### 2.5. Statistical Analysis

Data were assessed for normal distribution of quantitative variables, employing the Shapiro–Wilk test. If data showed normal distribution, we reported them as mean ± SD or as median (25^th^–75^th^ percentile) if they did not. Qualitative data were presented as frequencies and percentages. We compared the percentage of IPI between meals with Cochran's *Q* test and McNemar multiple tests with Bonferroni correction as post hoc. For comparing the proportion of participants within each BMI, diagnosed diseases, and IADL and ADL categories, we employed a *χ*^2^ goodness of fit test.

Thereafter, we performed a multinomial logistic regression to predict the risk of presenting middle or low scores (impaired functionality) in comparison with the group of high scores (reference) for both IADL and ADL when participants reported <30 g/meal (Model 1) or <0.4 g/kg/meal (Model 2). Both models were adjusted for IPI per day, sex, age, BMI categories, and number of diagnosed diseases. We reported the odds ratios (OR) for IPI per day and meal. We also reported the 95% CI for percentages and OR. All analyses were considered significant at a *p* value ≤0.05 and were carried out with the software SPSS® version 24 for Windows® (IBM Corp., Armonk, NY, USA).

## 3. Results

### 3.1. General Data

The mean values for age and height were 78.1 ± 8.2 years and 153.7 ± 9.1 cm, respectively. The median value for body weight was 62.0 [53.8–70.0] kg and 26.5 [22.8–29.6] kg/m^2^ for BMI. One hundred seven participants (56.3%, 49.2–63.2) had a BMI within the recommended range, 49 (25.8%, 20.1–32.5) below, and 34 (17.9%, 13.1–24.0) above it. These percentages showed significant differences (*χ*_2_^2^=46.94, *p* < 0.001). There were significant differences in diagnosed diseases groups, too (*χ*_2_^2^=16.62, *p* < 0.001). Thirty-seven participants (19.5%, 14.5–25.7) had no diagnosed diseases, 79 (41.6%, 34.8–48.7) had one diagnosed disease, and 74 (38.9%, 32.3–46.0) showed two or more diagnosed diseases. The most common diagnosed diseases were blood hypertension (*n*=100,52.6%), type two diabetes (*n*=67, 35.3%), osteoporosis (*n*=28, 14.7%), and chronic obstructive pulmonary disease (*n*=22, 11.6%).

### 3.2. Inadequate Protein Intake

**Figure 1:**
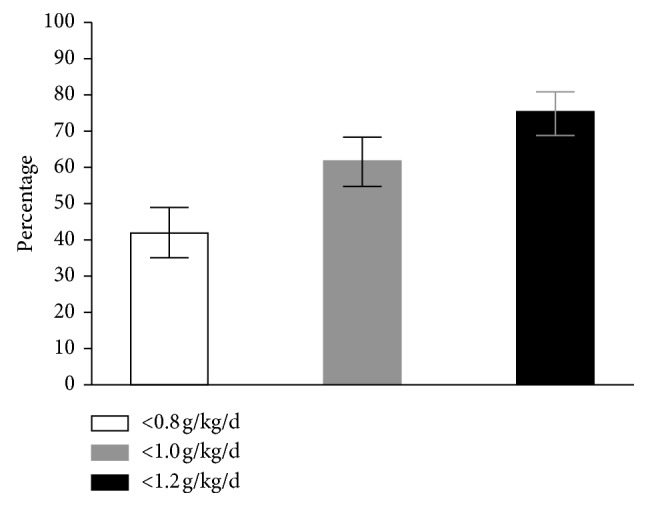
Inadequate protein intake per day in middle- to older-aged Mexican adults attending the Geriatrics Department at Western General Hospital, Zapopan, Jalisco, México (*n*=190). Bars represent the percentage of participants with inadequate protein intake per day for each cut point. Whiskers represent 95% confidence intervals. g/kg/d: grams of protein per kilogram of body mass per day.

Eighty participants (42.1%, 35.3–49.2) reported IPI per day for the protein RDA (<0.8 g/kg/d), 118 (62.1%, 55.0–68.7) reported a protein intake <1.0 g/kg/d, and 144 (75.8%, 69.2–81.3) reported eating <1.2 g/kg/d (Figure 1).

The highest percentage of IPI per meal was observed for dinner (≈93%) and breakfast (≈97%) when the 30 g protein/meal criterion was used. However, there were no significant differences between these two meals (*p* > 0.05). The lowest proportion was observed in lunch (≈66%), and it was significantly different from that of breakfast and dinner (Table 1). When IPI per meal was assessed with <0.4 g protein/kg criterion, dinner showed the highest percentage (≈92%), followed by breakfast (≈77%) and lunch (50%). However, in this case, all comparisons were significantly different (Table 1).

### 3.3. Functionality

For IADL, there was a higher proportion of participants with high scores, followed by low scores and middle scores; however, no significant differences were observed (*χ*_2_^2^=4.94, *p*=0.085). For ADL, the highest proportion was observed in participants with middle scores, followed by high scores and low scores; these comparisons reached statistical significance (*χ*_2_^2^=9.67, *p*=0.008) (Table 2).

### 3.4. Inadequate Protein Intake and Functionality

For IADL, no form of IPI was a significant variable in Model 1. However, IPI at dinner was a significant variable for IADL in Model 2. On the other hand, for ADL, IPI at lunch was a significant variable in Model 1, but no form of IPI was a significant variable in Model 2 (Table 3).

Age was the only significant covariate for IADL and ADL in both models (Table 3). As participants got older, there was higher risk for presenting middle (OR (95% CI), IADL: Models 1 and 2 = 1.11 [1.05–1.18]; ADL: Models 1 and 2 = 1.12 [1.06–1.18]) and low scores of functionality (IADL: Model 1 = 1.24 [1.15–1.32], Model 2 = 1.25 [1.17–1.35]; ADL: Models 1 and 2 = 1.27 [1.18–1.38]).

Models 1 and 2 explained about 40% of the variability of presenting middle or low functionality scores. However, they showed slightly higher Nagelkerke pseudo-*R*^2^ for ADL than IADL (Table 3). When the significant meal was removed from the models, the pseudo-*R*^2^ decreased slightly. Conversely, when age was removed from the models, the pseudo-*R*^2^ decreased importantly (Table 3).

For IADL, neither IPI per day nor per meal was significant risk factors for presenting middle nor low scores in comparison with the high score group in Model 1 (Table 4). However, in Model 2, the IPI at dinner (*p*=0.026) was the only IPI significant risk factor for presenting middle scores. The IPI per day and per meal was not significant risk factors for presenting low scores (Table 4).

For ADL, the IPI at lunch was the only IPI significant risk factor for presenting middle (*p*=0.043) and low (*p*=0.029) scores in Model 1 (Table 5). Conversely, in Model 2, neither IPI per day nor per meal were significant risk factors for presenting middle nor low scores (Table 5).

## 4. Discussion

The current study demonstrates that a high proportion of middle to older aged Mexican adults do not achieve the recommended protein intake per day (Figure 1) nor per meal (Table 1), and IPI at certain meals is related to impaired functionality in daily living activities (Tables 4 and 5).

While limited data exist regarding the habitual protein intake of Mexico's older population, Valenzuela et al. [27] reported that 37% of a sample of Mexican older adults did not reach the RDA for daily protein intake (0.8 g/kg/day). This result is similar to what we observed in this study (Figure 1). However, they did not report the proportion of older adults not achieving the recommended daily dose (1.0–1.2 g/kg/day) [20], which would be important as there is evidence suggesting older adults consuming protein below this recommendations have higher risk of undesired weight loss [40] and related frailty [41].

Regarding IPI per meal, Valenzuela et al. [27] reported that 81% and 86% of their sample consumed protein at a dosage smaller than 30 g at breakfast and dinner, respectively. These percentages are not so far from what we reported in this study for these two meals (Table 1). Therefore, we could confirm that a high proportion of middle to older aged Mexican adults do not fulfill the protein intake recommendations per meal at breakfast and dinner. To our knowledge, there is no other study reporting the IPI per day and per meal in Mexican older adults.

Studies in other countries reported that the proportion of older adults with IPI per day (<1.0 g/kg/day) varies widely, with 24% in the United Kingdom [30], 39% in the USA [28], and 61% in Germany [29]. In this study, we observed that the proportion of IPI with this cutoff point is similar to that reported in German older adults (Figure 1). This may be explained with data reported in previous studies, where protein intake patterns evaluated in German older adults look closer to those observed in Mexican adults [27, 29].

We did not find any study reporting the proportion of IPI with the cutoff point of <1.2 g/kg/day. We decided to use this ceiling because there is evidence suggesting that older adults consuming protein above this threshold have higher values of lean body mass [16, 18] and functionality [42]. However, we did not find any significant interaction between IPI per day (<1.2 g/kg/day) and impaired functionality (Tables 4 and 5). This lack of association might be related to the nature of the measure. That is, we obtained data about functionality in daily living activities from questionnaires and not directly measured, like gait speed, as was done in other studies that found an association [42].

Few studies are reporting IPI per meal. Cardon-Thomas et al. [30] reported that when employing the 0.4 g/kg/meal criterion, their sample of older adults showed 97% of IPI at breakfast, 58% at lunch, and 32% at dinner. These results differ from ours for breakfast and dinner but are similar for lunch using the same criterion (Table 1). Gingrich et al. [29] also suggest that consuming adequate amounts of protein per meal is not common in older adults. In their study, older adults consumed, on average, 0.72 meals per day with a protein dosage ≥0.4 g/kg, and about 55% of their sample did not consume more than two meals above this threshold for any day of the week. When the other criterion was used (<30 g/meal), Loenneke et al. [24] reported that ≈32% of their sample did not reach this protein amount for any meal, ≈52% only in one meal, and the remaining 16% at two or more meals, but they did not specify in which meal. We did not find any other study reporting IPI per meal with this criterion.

We observed that IPI at lunch (<30 g) was a significant risk factor for impaired functionality in ADL (Table 5) but not IADL (Table 4). IPI at lunch could be only associated with ADL but not IADL because the former consisted on more items than the later, leading to a broader assessment of different activities and therefore could be more sensitive to detect different functionality levels [37]. Similarly, while the cut points used for ADL were previously proposed according to their grade of dependence [36], those used for IADL were derived by dividing the scale into three equal ranges because we did not find any reference that proposed cut points related to its clinical importance.

We think IPI at lunch was associated with functionality because it is the meal where Mexican older adults have the opportunity to consume the highest amount of protein [27], and therefore, if they do not consume adequate amounts of protein in this meal, it would be less feasible they reach the adequate quantity in the other meals. Thus, high protein intake at lunch would be how Mexican older adults counteract the lack of protein intake in the other meals as compared with other countries, where the majority of protein is consumed during dinner [43, 44].

Conversely, it might be possible that protein intake per meal could exert a different mechanism over functionality other than reaching adequate daily protein intake. Previous studies have reported that older adults consuming ≥30 g protein per meal have higher values of leg strength and leg lean mass, even after adjusting for covariates, including total daily protein intake [24, 45]. Both strength and lean mass may be related to higher functionality [8, 31, 46]. However, Buckner et al. [45] evaluated protein intake per meal (two 24 h dietary recalls), leg lean mass with dual-energy X-ray absorptiometry, and leg strength with an isokinetic dynamometer in middle to older aged adults (50–85 y). They suggest that consuming ≥30 g of protein at dinner could be more advantageous than consuming it at lunch for higher values of leg strength and leg lean mass in this population [45]. Nonetheless, we did not find that IPI at dinner would be a significant risk factor for impaired functionality when we employed the 30 g/meal criterion (Tables 4 and 5). Conversely, we found that IPI at dinner was a significant risk factor for impaired functionality when we used the 0.4 g/kg/meal criterion and for IADL middle scores only (Table 4). Again, the differences between these studies may be related to the different nature of the outcome variable (a direct measure of strength and lean mass vs. self-reported information about functionality).

The models reported here explained about 40% of the variability in the presence of impaired functionality. Most of this variability was explained by age and a small proportion by IPI per meal (Table 3) possibly because there are other nondietary (e.g., physical activity and exercise) and dietary (e.g., protein sources and antioxidant intake) factors also related with strength, lean mass, and functionality in older adults [47]. Nonetheless, when we compared IPI at lunch and dinner, both are significant risk factors for different forms of functionality and have almost the same contribution to their respective model (Table 3). However, IPI at lunch would be a more likely risk factor because it was significant for both middle and low scores (Table 5) and dinner for middle but not low scores (Table 4).

To our knowledge, this is the first study to analyze the association between IPI per day and per meal with daily living functionality in middle to older aged adults. However, some studies reported that low consumption of protein (among other nutrients) is a risk factor for developing frailty [48] or showing low lean body mass [18, 49, 50], strength [49] or functionality [42]. This may be a vicious circle, as the lack of functionality is a risk factor for low protein intake [51]. In complement, there should be more research in other ways to assess daily living functionality, like physically active and sedentary time [30], along with protein intake assessment to amplify our knowledge about their possible interaction.

Even though we have to be cautious when interpreting these results, there are limitations to our data. Firstly, we are aware that nutrient intake assessed by 24 hour dietary recalls is highly variable and would not be representative of the actual diet [39, 52]. Therefore, further research should address dietary protein intake with more representative dietary assessment tools (e.g., food diary). Secondly, the functionality assessment relied on self-reported information, which also would be prone to misreporting [34]. Additionally, ceiling effect would be a limitation as 18.9%, and 20.5% of our sample reported the highest score of the IADL and ADL scales, respectively, and ceiling effect is considered when this happens for >15% of the sample [53]. Thus, we consider that objective measurement tools should be used along these instruments to overcome the possible ceiling effect in the future. Nevertheless, these questionnaires have reported high reliability to gather information about daily living activities [37, 38, 54, 55]. Thirdly, our sample consisted of middle to older aged adults attending a tertiary care hospital for their habitual or initial medical screening. Furthermore, most of them reported at least one diagnosed disease, and about half of them had a BMI out of the normal range. Therefore, these results may differ in other healthier samples [29]. Fourthly, there could be a sample selection bias, as the nonincluded participants could show more physical and mental limitations as suggested for the inclusion criteria. Finally, despite our sample size being larger than that reported by Valenzuela et al.'s study (190 vs. 79, respectively) [27], it was still low as was the number of participants with sufficient protein intake per day and per meal (Figure 1, Table 1), which may affect the statistical analyses as observed in wide 95% CI for OR. Similarly, this limitation impeded us to calculate OR for IPI at dinner in Model 1, which may lead to an improper adjustment in the model (Table 5). Again, larger sample size could overcome this limitation.

## 5. Conclusion

In summary, our data suggest that IPI per meal is high in middle- to older-aged Mexican adults and, at specific meals, it is a significant risk factor for impaired functionality in activities of daily living, even after adjusting for confounding variables. Further research should aim to develop strategies to overcome this issue and find the possible motivations in older adults that may lead to more feasible nutritional interventions [56].

## Figures and Tables

**Table 1:** Inadequate protein intake per meal in middle- to older-aged Mexican adults attending the Geriatrics Department at the Western General Hospital, Zapopan, Jalisco, México (*n*=190).

	*n*	%	(95% CI)
Women	141	74.2	(67.6–79.9)

IPI per meal (<30 g)			
Breakfast	176	92.6	(88.0–95.6)^a^
Lunch	126	66.3	(59.3–72.7)^b^
Dinner	184	96.8	(93.3–98.5)^a^

IPI per meal (<0.4 g/kg)			
Breakfast	147	77.4	(70.9–82.7)^a^
Lunch	95	50.0	(43.0–57.0)^b^
Dinner	175	92.1	(87.4–95.2)^c^

Different letters denote significant differences (*p* < 0.05) for IPI percentages between meals within the same criterion. IPI: inadequate protein intake.

**Table 2:** Distribution of participants according to their obtained functionality score (*n*=190).

	*n*	%	(95% CI)	*p*
Instrumental activities of daily living				
High scores	76	40.0	(33.3–47.1)	0.085
Middle scores	51	26.8	(21.0–33.6)	
Low scores	63	33.2	(26.9–40.1)	

Activities of daily living				
High scores	63	33.2	(26.9–40.1)	0.008
Middle scores	81	42.6	(35.8–49.7)	
Low scores	46	24.2	(18.7–31.1)	

**Table 3:** Summary of the significance of independent variables introduced in the multinomial logistic regression for estimating functionality.

	Functionality			
	IADL^a^		ADL^a^	
	Model 1	Model 2	Model 1	Model 2
Variables in the model^b^	*p* values		*p* values	
IPI day	0.42	0.24	0.40	0.82
IPI breakfast	0.15	0.77	0.75	0.29
IPI lunch	0.16	0.16	0.044	0.47
IPI dinner	0.51	0.035	0.17	0.95
Age	0.001	0.001	0.001	0.001
Sex	0.35	0.42	0.63	0.47
BMI categories	0.99	0.77	0.45	0.52
Diagnosed diseases	0.75	0.88	0.51	0.63

Model type	Pseudo-*R*^2f^		Pseudo-*R*^2^	
Full model^c^	0.379	0.382	0.426	0.402
Without the significant meal^d^	NA	0.356	0.402	NA
Without age^e^	0.141	0.114	0.204	0.148

^a^Outcome variable. ^b^Independent variables. ^c^Included all listed variables. ^d^Included all variables except the IPI at the meal with a significant *p* value. ^e^Included all variables except the age. ^f^Nagelkerke pseudo-*R*^2^. ADL: activities of daily living; IADL: instrumental activities of daily living; IPI: inadequate protein intake; IPI day: inadequate protein intake per day (<1.2 g/kg/d); Model 1: inadequate protein intake per meal considered as <30 g protein/meal; Model 2: inadequate protein intake per meal considered as <0.4 g protein/kg/meal; NA: not applicable.

**Table 4:** Risk of impaired functionality for instrumental activities of daily living (IADL) related to the inadequate protein intake per day and per meal in middle to older aged Mexican adults (*n*=190).

	Model 1				Model 2			
	IPI per day (<1.2 g/kg)	IPI per meal (<30 g)			IPI per day (<1.2 g/kg)	IPI per meal (<0.4 g/kg)		
		Breakfast	Lunch	Dinner		Breakfast	Lunch	Dinner
High scores		1 (reference)				1 (reference)		

Middle scores	0.47 (0.14–1.56)	7.70 (0.70–84.97)	1.83 (0.73–4.57)	3.25 (0.29–36.42	0.39 (0.11–1.37)	1.20 (0.41–3.49)	2.05 (0.83–5.10)	7.64^*∗*^ (1.27–45.85)

Low scores	0.51 (0.14–1.85)	1.81 (0.31–10.61)	2.59 (0.94–7.15)	3.11 (0.24–33.83)	0.36 (0.09–1.44)	1.52 (0.48–4.74)	2.31 (0.87–6.10)	4.74 (0.92–24.47)

Data expressed as odds ratios (95% confidence intervals). Models were adjusted for sex, age, BMI categories, and number of diagnosed diseases. IPI: inadequate protein intake; Model 1: inadequate protein intake per meal considered as <30 g protein/meal; Model 2: inadequate protein intake per meal considered as <0.4 g protein/kg/meal. ^*∗*^Significant association (*p* ≤ 0.05).

**Table 5:** Risk of impaired functionality for activities of daily living (ADL) related to the inadequate protein intake per day and per meal in middle to older aged Mexican adults (*n*=190).

	Model 1				Model 2			
	IPI per day (<1.2 g/kg)	IPI per meal (<30 g)			IPI per day (<1.2 g/kg)	IPI per meal (<0.4 g/kg)		
		Breakfast	Lunch	Dinner		Breakfast	Lunch	Dinner
High scores		1 (reference)				1 (reference)		

Middle scores	1.08 (0.34–3.38)	0.68 (0.11–4.23)	2.40^*∗*^ (1.03–5.62)	2.38 (0.25–22.32)	1.47 (0.45–4.84)	0.80 (0.28–2.32)	1.63 (0.71–3.74)	1.26 (0.30–5.37)

Low scores	0.47 (0.12–1.89)	0.45 (0.05–3.80)	3.82^*∗*^ (1.15–12.65)	—^§^	1.37 (0.30–6.16)	0.40 (0.11–1.37)	1.16 (0.39–3.45)	1.15 (0.19–6.87)

Data expressed as odds ratios (95% confidence intervals). Models were adjusted for sex, age, BMI categories, and number of diagnosed diseases. IPI: inadequate protein intake; Model 1: inadequate protein intake per meal considered as <30 g protein/meal; Model 2: inadequate protein intake per meal considered as <0.4 g protein/kg/meal. ^*∗*^Significant association (*p* ≤ 0.05). ^§^Unable to calculate it due to small sample size in this category.

## Data Availability

The data used to support the findings of this study are available from the corresponding author upon reasonable request.
